# Prevalence and Antimicrobial Resistance Patterns of Hospital Acquired Infections through the COVID-19 Pandemic: Real-Word Data from a Tertiary Urological Centre

**DOI:** 10.3390/jcm12237278

**Published:** 2023-11-24

**Authors:** Filippo Gavi, Barbara Fiori, Carlo Gandi, Marco Campetella, Riccardo Bientinesi, Filippo Marino, Daniele Fettucciari, Francesco Rossi, Stefano Moretto, Rita Murri, Francesco Pierconti, Marco Racioppi, Emilio Sacco

**Affiliations:** 1 Postgraduate School of Urology, Università Cattolica del Sacro Cuore, Largo Francesco 6 Vito 1, 00168 Rome, Italy; 2 Department of Urology, Fondazione Policlinico Universitario Agostino Gemelli IRCCS, Largo Francesco 8 Vito 1, 00168 Rome, Italy; 3 Department of Infectious Disease, Fondazione Policlinico Universitario Agostino Gemelli IRCCS, Largo Francesco 8 Vito 1, 00168 Rome, Italy; 4 Department of Pathology, Fondazione Policlinico Universitario Agostino Gemelli IRCCS, University of Sacred Heart, 00168 Rome, Italy; 5 Urology Department, Isola Tiberina—Gemelli Isola Hospital, Catholic University Medical School, 00168 Rome, Italy

**Keywords:** antimicrobial stewardship, urinary tract infection, antibiotic resistance

## Abstract

Background: Antimicrobial resistance (AMR) remains a significant public health concern, closely linked to antibiotic overuse. During the COVID-19 pandemic, broad-spectrum antibiotics were frequently administered, potentially exacerbating AMR. This study aimed to assess AMR patterns in our urology department before and after the pandemic. Methods: The study encompassed patients admitted to our urology department from January 2016 to December 2022, with confirmed urinary tract infection, bloodstream infection, or wound infection based on positive culture results. Descriptive statistics, including mean, frequency, and percentage, summarized the data. Trends were analyzed using the Joinpoint Regression program. Results: A total of 506 patients were included. *Escherichia coli* and *Klebsiella pneumoniae* displayed resistance rates of 65% and 62% to ciprofloxacin, respectively. *K. pneumoniae* showed resistance rates of 41% to piperacillin tazobactam and 3rd generation cephalosporins (3GC). Carbapenem resistance was observed in 38% of *K. pneumoniae* isolates. Additionally, 26% of *E. coli*, 26% of *K. pneumoniae*, and 59% of *Proteus mirabilis* isolates were ESBL-positive. Among gram+, 72% of *Staphylococcus aureus* isolates were *MRSA*, and 23% of *Enterococcus faecium* isolates were *VRE*. Trends in antimicrobial susceptibility patterns over the 7-year study period revealed a statistically significant decrease in *E. coli* resistance to amoxicillin-clavulanic acid (APC: −5.85; C.I. 95% *p* < 0.05) and a statistically significant increase in *K. pneumoniae* resistance to 3GC (APC: 9.93; CI (−19.9–14.4 95% *p* < 0.05). There were no statistically significant differences in AMR incidence pre- and post-COVID-19. Conclusion: The COVID-19 pandemic did not appear to influence the AMR incidence in our urology department. However, the overall prevalence of AMR and MDROs in our department remains high compared to European AMR.

## 1. Introduction

Healthcare-associated infections (HAIs) are a global health issue [1]. The prevalence rate of HAIs ranges from 3.0% to 20.7%, with an incidence rate of 5% to 10% [2]. These infections contribute to increased morbidity, mortality, and a significant economic burden [3]. Among HAIs, urinary tract infections (UTIs) are the most encountered at urological departments [4,5]. Due to the frequent and inappropriate use of antimicrobial medications and the inadequacy of regimens, antimicrobial resistance (AMR) is a naturally occurring evolutionary process in bacteria that is exacerbated by selection pressure [6]. Additionally, bacteria that are drug-resistant might spread the resistance to different bacterial genera or species [7,8].

In Italy, there has been an increase in cases involving multidrug-resistant organism (MDRO). The epidemiology of MDRO infections differs by department, hospital, geographic region, and year [8,9]. These bacteria have developed resistance to multiple classes of antibiotics, rendering treatment more challenging and increasing the risk of severe infections and mortality [10]. The overuse and misuse of antibiotics play a substantial role in the emergence of MDRO. Therefore, efforts are being made to reduce unnecessary antibiotic usage and improve infection control measures [2,11]. Presently, MDRO represent a significant public health concern, with the most common pathogens being *methicillin-resistant Staphylococcus aureus* (*MRSA*), *vancomycin-resistant enterococcus* (*VRE*), extended-spectrum cephalosporin resistance in Enterobacteriaceae indicative of extended-spectrum beta-lactamase (*ESBL*) production, carbapenem-resistant Enterobacteriaceae, carbapenem-resistant *Acinetobacter* spp., and MDRO *Pseudomonas aeruginosa* [12]. To address these challenges, our hospital has developed various protocols to reduce antibiotic misuse. Our efforts in the urology department focus on implementing fast diagnostic tools and maintaining high clinical standards to ensure responsible and appropriate antibiotic use.

During the pandemic, congestion in hospitals and excessive antimicrobial usage in COVID-19 patients most likely hastened the emergence and spread of AMR [13,14]. Depending on the healthcare system and public health policies in each country, the impact of COVID-19 on AMR differed greatly. The most often prescribed broad-spectrum antibiotics in hospitals were azithromycin, amoxicillin-clavulanic acid, and levofloxacin, and they were given to about two third of COVID-19 patients [15]. However, several studies and/or review articles have examined the prevalence of MDRO bacteria and the changes in the use of antibiotics prior to and during the COVID-19 pandemic [16,17,18]. This study aimed to evaluate the prevalence of HAIs and antimicrobial susceptibility patterns in a tertiary urological centre and to provide updated real-word data before and after the COVID19 pandemic for the development of institutional programs aimed at enhancing antimicrobial stewardship.

## 2. Materials and Methods

Study Design and Setting. This cross-sectional study was conducted at the Urology Department of Policlinico Agostino Gemelli Hospital, Rome, Italy, over a period of 7 years from January 2016 to December 2022. Of all patients with clinically suspected infection, only patients admitted for at least 48 h at our urology department with a culture-proven UTI, blood stream infection (BSI) or surgical site infection (SSI) were included in the study. Clinical suspicion of infection was based on signs such as high temperature (>38 °C), chills, hypotension, surgical wound redness, delayed healing, pain, or tenderness. Leukocytosis and inflammatory markers C-reactive protein (CRP) and procalcitonin (PCT)) were also considered in the diagnostic algorithm. Urine culture in case of a suspected UTI (lower urinary tract symptoms with pelvic pain, cloudy or strong-smelling urine, hematuria), a blood culture in case of suspected sepsis (fever, confusion or disorientation, hypotension, leukocytosis or leucopenia, systemic symptoms like high heart rate and shortness of breath, low blood pressure, clammy or sweaty skin), and a wound culture in case of wound infection (pus, spreading redness, increased pain or swelling) were performed. Antibiogram were performed to all samples. Exclusion criteria included age < 18 years and missed data on antibiograms reports. Midstream urine, blood, and wound specimens, collected from patients, were subcultured on a set of selective and nonselective routine agar plates and incubated under appropriate atmospheric conditions for 24 h or re-incubated for 48 h as necessary. Bacterial isolates were identified by VITEK 2 (bioMérieux, Marcy l’Etoile, France) (from 2007 to 2009) and matrix-assisted laser desorpt ionionization-time of flight (MALDI-TOF) mass spectrometry (MALDI BioTyper, Bruker Daltonik GmbH, Leipzig, Germany). Antimicrobial susceptibility testing of the bacterial isolates was performed as part of the routine analyses with the Vitek 2 (bioMérieux) and/or Etest (bioMérieux) and interpreted according to EUCAST breakpoints [19].

Data Analysis and Interpretation. The data were entered and analyzed using STATA/MP 17.0. Descriptive statistics including mean with standard deviation, frequency and percentage were used to summarize the data and presented in the form of texts, table, and graphs as appropriate.

To analyze the trends of the annual antimicrobial resistance rate from 2016 to 2022, we used the Joinpoint Regression program, version 4.6.0.01. Time trend analyses were conducted for the AMR for *E. coli* and *K. pneumoniae* during the seven years of the study. By dividing the data into time periods before and after 2020, Joinpoint Regression made it possible to pinpoint the years in which the trend changed statistically significantly. The methodology assessed if the annual percentage change (APC) in prevalence is statistically different from zero for each time segment (Segment 1 2016–2020 and segment 2 2020–2023) and estimates the APC for that period.

## 3. Results

Baseline Characteristics. Table 1 displays the patients’ baseline characteristics by type of infection. A total of 506 patients were included in the study, 331 (65%) were male and 175 (35%) were female. The mean age of study participants was 68.5 years (SD 13.41, IQR 20–92 years).

**Figure 1:**
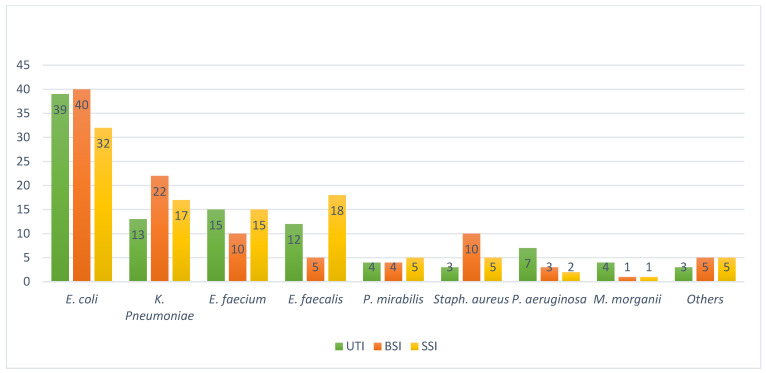
Distribution of bacteria (n) isolated at the Urology department from 2016 to 2022 by type of infection. Note: UTI: urinary tract infection; BSI: blood stream infection; SSI: surgical site infection.

Prevalence of infections. A total of 506 tests resulted positive for infection. In Figure 1 are reported the bacteria distribution rates by type of infection.

**Figure 2:**
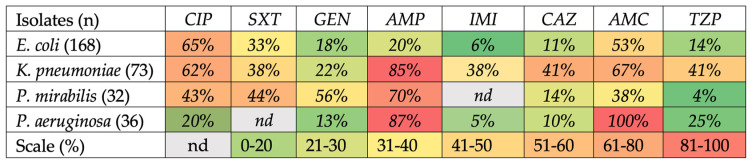
Heatmap of Gram-negative bacteria antibiotic resistance. Note: AMC: amoxicillin-clavulanic acid; AMP: ampicillin; CAZ: ceftazidime; CIP: ciprofloxacin; GEN: gentamicin; IMI: imipenem; SXT: cotrimoxazole; TZP: piperacillin plus tazobactam; nd: not detected.

Antimicrobial Susceptibility. The antimicrobial susceptibility of the most frequently encountered Gram-negative isolates is shown in Figure 2. Among the isolates, 65% of *E. coli,* 62% of *K. pneumoniae*, 43% of *P. mirabilis* and 20% of *P. aeruginosa* were resistant to ciprofloxacin; 33% of *E. coli*, 38% of *K. pneumoniae* and 44% of *P. mirabilis* were resistant to cotrimoxazole; 18% of *E. coli*, 22% of *K. pneumoniae*, 56% of *P. mirabilis* and 13% of *P. aeruginosa* were resistant to gentamicin; 20% of *E. coli*, 85% of *K. pneumoniae*, 70% of *P. mirabilis* and 87% of *P. aeruginosa* were resistant to ampicillin; 6% of *E. coli*, 38% of *K. pneumoniae* and 5% of *P. aeruginosa* were carbapenem resistant; 11% of *E. coli, 41% of K. pneumoniae, 14% of P. mirabilis* and 10% of *P. aeruginosa* were resistant to ciprofloxacin; 53% of *E. coli*, 67% of *K. pneumoniae*, 38% of *P. mirabilis* and 100% of *P. aeruginosa* were resistant to amoxicillin-clavulanic acid; 14% of *E. coli*, 41% of *K. pneumoniae,* 4% of *P. mirabilis* and 25% of *P. aeruginosa* were resistant to piperacillin plus tazobactam. Among isolated pathogens, 43 (26%) *E. coli*, 19 (26%) *K. pneumoniae* and 19 (59%) *P. mirabilis* isolates were ESBL-producers. Among the *E. coli* isolates, 10 (9.9%) were resistant to three different antibiotic classes β-lactams (penicillins, penicillins with β-lactamases inhibitors, cephalosporins), aminoglycosides and fluoroquinolones.

**Figure 3:**
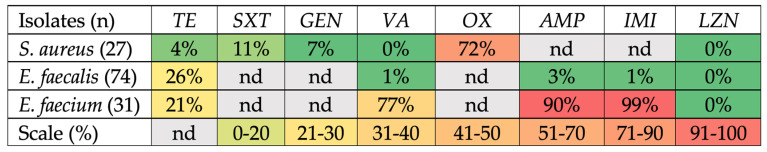
Heatmap of Gram-positive bacteria antibiotic resistance. Note: AMP: ampicillin; GEN: gentamicin; IMI: imipenem; LZN: linezolid; SXT: cotrimoxazole; TE: teicoplanin; VA: vancomycin; OX: Oxacillin; nd: not detected.

The antimicrobial susceptibility of the most frequently encountered Gram-positive isolates is shown in Figure 3. Among the isolates, 4% of *S. aureus*, 26% of *E. faecalis* and 21% of *E. faecium* were resistant to teicoplanin; 7% of *S. aureus* were resistant to cotrimoxazole; 0% of *S. aureus*, 1% of *E. faecalis* and 77% of *E. faecium* were resistant to vancomycin; 3% of *E. faecalis* and 90% of *E. faecium* were resistant to ampicillin; 1% of *E. faecalis* and 99% of *E. faecium* were resistant to imipenem; no AMR were found to linezolid. Among the *S. aureus* isolates, 19 (72%) were *MRSA*. Among *Enterococcus faecium* 24 (77%) isolates were *VRE*. *Carbapenem-resistant Acinetobacter baumannii* (*CR-Ab*) was isolated in four patients.

**Figure 4:**
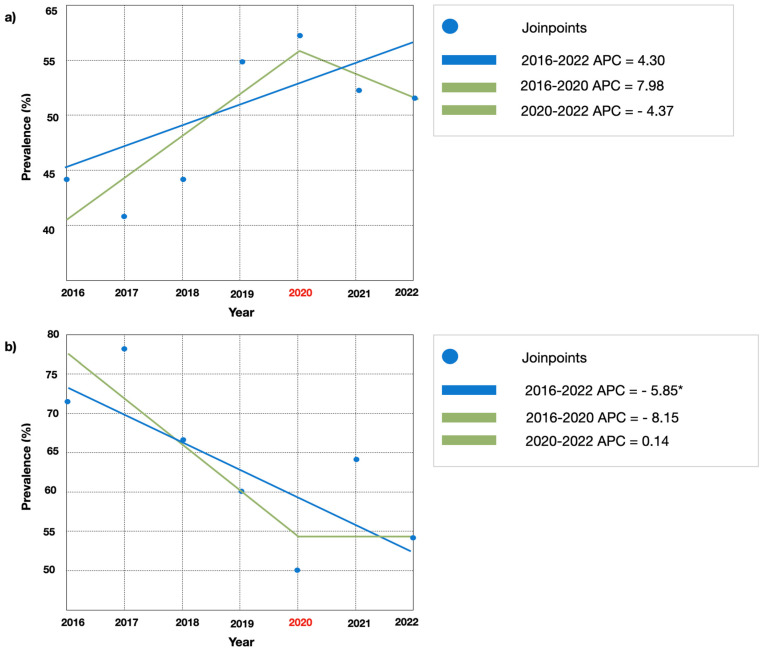
(**a**) Joinpoint regression for *E. coli* antimicrobial resistance to ciprofloxacin. (**b**) Joinpoint regression for *E. coli* antimicrobial resistance to amoxillin plus clavulanic acid. Note: APC: annual percentage change; *: *p* < 0.05.

**Figure 5:**
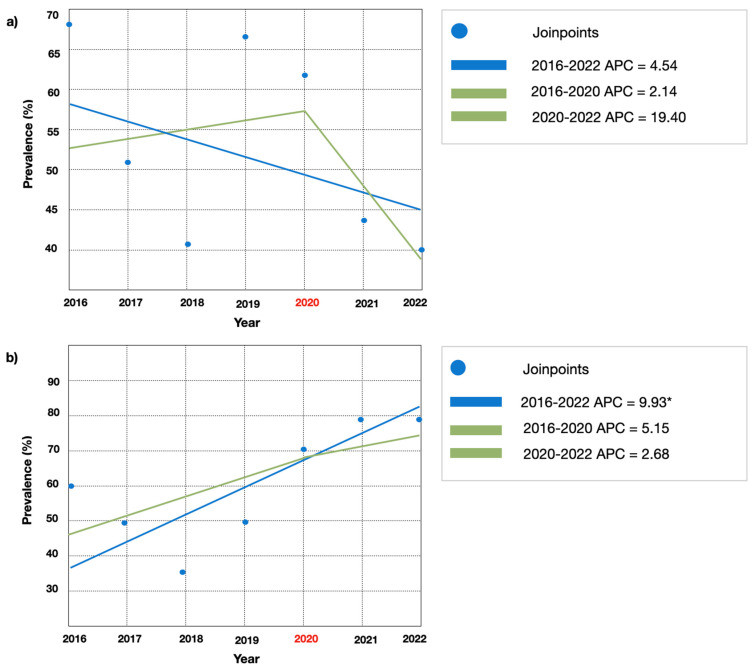
(**a**) Joinpoint regression for *K. pneumoniae* antimicrobial resistance to amoxicillin-clavulanic acid. (**b**) Joinpoint regression for *K. pneumoniae* antimicrobial resistance to ceftazidime. Note: APC: annual percentage change; *: *p* < 0.05.

Seven years antimicrobial susceptibility trend of predominant pathogens. The seven-year trend analysis showed a non-significant APC change for *E. coli* and *K. pneumoniae* antimicrobial resistance rates, with the exception for amoxicillin-clavulanic acid (APC: −5.85; C.I. 95% *p* < 0.05) and ceftazidime (APC: 9.93; CI (−19.9–14.4 95% *p* < 0.05), respectively. Trends analysis showed a non-significant difference in AMR between pre and post COVID19 pandemic for the most frequent isolates (Escherichia coli and Klebsiella pneumoniae). Figure 4 displays AMR trends to ciprofloxacin and amoxicillin-clavulanic acid for *E. coli.* Figure 5 displays AMR to amoxicillin-clavulanic acid and to ceftazidime for *K. pneumoniae*.

## 4. Discussion

*E. coli* was the most isolated pathogen for UTI followed by *K. pneumoniae* and *Enterococcus* spp., mirroring the results of the Global Prevalence Study of Infections in Urology [20]. *E. coli* and *K. pneumoniae* showed high resistance to ciprofloxacin, cotrimoxazole, amoxicillin-clavulanic acid. Resistance to ciprofloxacin in *E. coli* and *K. pneumoniae* was as high as 65% and 62%, respectively. These results were consistent with the 2020 WHO report on antibiotic resistance in Europe [2]. These data are of great concern showing an increase in the ciprofloxacin resistance rate, higher than the data collected by the European Centre for Disease Prevention and Control reporting a mean resistance rate in Italy for *E. coli* and *K. pneumoniae* of 37.6% and 52.4%, respectively. Resistance to third generation cephalosporins and carbapenems generally was higher in *K.* pneumoniae than *E. coli*. Third-generation cephalosporin resistance in *K. pneumoniae* was 41%. The mean antimicrobial resistance rate to carbapenems in Italy in 2021 for *K. pneumoniae* was 26.7%, in Europe instead between 0.6 and 6%. Our data confirmed this dramatic difference. Overall, most of the isolates had higher antimicrobial resistance rates compared to Europe and Italy rates [21]. Almost 60% of *K. pneumoniae* isolates were ESBL+, implying a prolonged antibiotics therapy, more serious infections and longer hospital stays. Since 2010, there have been numerous studies on the distribution of ESBLs in the population, and there are no evidence of a decline in the dissemination of ESBL-producing bacteria globally [22]. The prevalence of *MRSA* in our study was 20% higher than expected based on the data of the 2020 WHO report on antibiotic resistance in Italy [2]. *MRSA* is of great concern because it is associated with high rates of clinically relevant infection, increased hospital stay and cost, greater mortality and high vancomycin usage [23]. Several modeling studies have predicted that the prevalence of these organisms will increase rapidly in the absence of intervention. Italy has implemented several initiatives to address this issue, including the establishment of a national surveillance system for antibiotic resistance and the promotion of responsible antibiotic use in healthcare settings [24].

Seven-year trends for *E. coli* showed a significant increase in AMR for amoxicillin-clavulanic acid. Amoxicillin-clavulanic acid is a broad-spectrum antibiotic, and it is one of the most prescribed antibiotics, but there are regional differences in the amount of *E. coli* resistance to amoxicillin-clavulanic acid. For this reason, the local susceptibility of *E. coli* should serve as the basis for the empiric regimens for simple and complex UTIs. However, when available, susceptibility results should be used to establish final regimens [25].

A worrisome increase in *K. pneumoniae* resistance to *3GCR* and CR globally was reported by Uria et al. When treating infections caused by Gram-negative bacteria that are resistant to many drugs, carbapenem frequently serves as the final line of treatment due to its broad spectrum of action and stability against a variety of inhibitors. Trends projections of AMR suggested that over half of *K. pneumoniae* isolates will be CR by 2030 [26]. According to EARS-Net, various nations have observed rising rates of bloodstream infections caused by *3GCR K. pneumoniae.* Moreover, a combination resistance to multiple antibiotic groups is a common occurrence, with over half of the *E. coli* isolates and over a third of the *K. pneumoniae* isolates showing resistance to at least one antimicrobial group under monitoring.

AMR percentages in antimicrobial groups that were tracked for both species were typically greater in *K. pneumoniae* than in *E. coli*. [21]. No trend analysis was performed for *ESBL+*, *MRSA*, *VRE*, *3GCR* and *CR-Ab* because of the small number of isolates. Data are not unanimous, although the majority of articles in the literature reported an increase trend in the prevalence of *VRE*, *MRSA* and *CR-Ab* [18,27,28]. In our study, 77% of *E. faecium* isolates were *VRE*. The prevalence of *VRE* colonization in different patient groups was investigated in nine cross-sectional studies, two cohort studies and one pre-post study in hospitals in Europe [29]. The prevalence ranged between 1.2% and 27.7% [21]. *VRE* continues to be a serious issue in healthcare due to the few available treatment options and rising prevalence of *VRE* in Europe. Effective strategies to control the spread of *VRE* are required [29].

To the best of our knowledge, this study represents the first report on AMR within a Urology department pre- and post- COVID-19 pandemic. In this study, no significant changes were observed in the frequency of AMR rates before and after the COVID-19 pandemic. Similar results were reported by Segala et al. [30], no differences were found in the incidence of *3GCR* BSI during the COVID-19 period compared to a pre-COVID-19 cohort. A retrospective study conducted in Italy between 2017 and 2020 showed a significant reduction of infections by MDRO for the period between March and June 2020 [31] and in a monocentric study conducted in Spain the incidence of hospital-acquired MDRO had a stable trend. Lazio is one of the areas with the highest AMR rates in Italy [24]. No clear causes have been reported to these high AMR rates, although AMR is strictly associated with excessive use of antibiotics, absence of proper surveillance system, inadequate infection control practices in hospital, limitation of recent AMR data and lack of awareness [32].

The implementation of COVID-19 protocols in our department did not appear to affect the AMR and MDRO rates. Nevertheless, the existing literature suggests that MDRO frequency increased during the COVID-19 pandemic, particularly for carbapenem-resistant Klebsiella pneumoniae, *VRE* and *MRSA* particularly in ICUs [18,33,34,35]. This knowledge gap necessitates further investigation to understand the impact of COVID-19 on AMR dynamics in these settings. It is important to consider the differences in patient populations, surgical procedures, and antibiotic usage between ICUs and surgical departments. Patients in ICU departments had higher rates of bacterial coinfections during the COVID-19 pandemics which can explain the higher rates of MDRO in this setting. At the same time, the COVID-19 pandemic was characterized by a slowdown in detection and reporting in surveillance programs, with fewer samples being received and analysed, and a rise in clinical samples, particularly in high-income nations. As a result, it is crucial to use caution when analyzing these data because there could be biases caused by changes in the patients and testing denominators that are not typical [18]. These factors may contribute to variations in AMR rates pre and post COVID-19, warranting context-specific interventions and surveillance strategies. The implementation of antibiotic stewardship protocols that incorporate actions to prevent infections and control MDRO spread have already demonstrated to cause an important decrease in the number of infections and deaths from antibiotic-resistant infections [36]. Therefore, updated real-world data are still essential to program precise and concrete actions to enhance antibiotic stewardship protocols. At our Urology department, we implemented a stewardship protocol in cooperation with our infection disease specialists. By constantly analyzing the microbial susceptibility patterns and trends, we can administer an effective empiric antibiotic therapy in more than 90% of the responsible microorganisms.

According to our results, the use of Piperacillin/tazobactam 4.5 g intravenously every 6 h is effective against most bacteria isolated at our urology department. If sepsis signs are present, our infectious disease consultant is called to decide the best course of action. Depending on the patient’s clinical conditions the most appropriate antibiotic will be administered. In case of previous MDRO colonization our infectious disease consultant is promptly called and if necessary, more effective antibiotics are administered.

The use of the MALDI BioTyper system coupled to the FilmArray BCID panel for the direct detection/identification of the causative organism of BSI gives us the opportunity to start a precise and specific antibiotic therapy very quickly, avoiding unnecessary empiric antibiotic therapies [37].

Once the blood cultures are positive, we can identify the microorganism in 1 h and to have a complete antibiogram in 24 h. NG-Test Carba 5 Assay for Direct Blood Culture Testing assures a fast and precise detection of carbapenemase-producing enterobacteria (CPE) infections, especially for *K. pneumoniae*, an endemic pathogen in our region. The constant collaboration between the microbiology laboratory, the department of infectious diseases and the department of urology permits us to spare long days of empiric antibiotics therapies and to be more effective and limit the surge of antibiotic resistance.

This study had some limitations. Firstly, the number of the isolates were limited, therefore the susceptibility trends per year were considered only for the most frequent uropathogens (*E. coli* and *K. pneumoniae*). Secondly, this study is monocentric therefore our data should be interpreted and used with caution.

Nonetheless, our study showed that AMR is a real and worrisome issue. Our data will help to implement local AMR surveillance programs and to update antibiotic stewardship programs.

## 5. Conclusions

In conclusion, the COVID-19 pandemic did not influence the AMR incidence in our urology department. Overall AMR and MDROs prevalence in our department is high compared to data from Europe. It is essential to maintain a strict surveillance of infections and to reinforce an efficient use of antibiotics limiting the spreading of MDRO is pivotal.

## Figures and Tables

**Table 1:** Patients baseline characteristics by type of infection.

	UTI	BSI	SSI	Total
Mean age years, (sd)	67 (13)	71 (10)	69 (10)	69 (10)
Sex n, (%)				
Male	179 (64%)	89 (69%)	63 (72%)	331 (65)
Female	110 (36)	40 (31)	25 (28)	175 (35)
Years n, (%)				
2016	30 (54)	9 (16.3)	16 (29)	55 (100)
2017	24 (50)	16 (33.3)	8 (16.6)	48 (100)
2018	25 (41)	17 (28)	19 (31)	61 (100)
2019	25 (41)	15 (24.5)	21 (34)	61 (100)
2020	28 (53)	21 (39.6)	4 (7.5)	53 (100)
2021	56 (54)	33 (32)	14 (13.5)	103 (100)
2022	36 (60)	18 (30)	6 (10)	60 (100)
Total	289 (100)	129 (100)	88 (100)	506 (100)

UTI: urinary tract symptoms; BSI: blood stream infection; SSI: surgical site infection; sd: standard deviation.

## Data Availability

The data presented in this study are available on request from the corresponding author.
